# RETRACTED ARTICLE: *ABCB1* (1199G>A) polymorphism regulates the efficacy of docetaxel and imatinib mesylate in HEK293 recombinant cell lines

**DOI:** 10.1007/s00280-015-2802-z

**Published:** 2015-06-21

**Authors:** Rui Peng, Hong Zhang, Ying Zhang, Dan-yun Wei

**Affiliations:** Department of Pharmacy, Renmin Hospital of Wuhan University, Wuhan, 430060 China

The Editors-in-Chief of Cancer Chemotherapy & Pharmacology have decided to retract this article. Substantial portions of this article were found to have been published previously by: G. Dessilly, L. Elens, N. Panin, A. Capron, A. Decottignies, JB Demoulin V. Haufroid, ABCB1 1199G (2014) A genetic polymorphism (Rs2229109) influences the intracellular accumulation of tacrolimus in HEK293 and K562 recombinant cell lines. PLoS One. 2014 Mar 12;9(3):e91555. The authors apologize to the Editors and readers, as well as to the authors of the original article.

